# Neurochemistry of Visual Attention

**DOI:** 10.3389/fnins.2021.643597

**Published:** 2021-05-05

**Authors:** Denise Elfriede Liesa Lockhofen, Christoph Mulert

**Affiliations:** Center for Psychiatry and Psychotherapy, Justus-Liebig University, Hessen, Germany

**Keywords:** visual attention, attentional selection, neurochemistry, psychopharmacology, neuromodulator

## Abstract

Visual attention is the cognitive process that mediates the selection of important information from the environment. This selection is usually controlled by bottom-up and top-down attentional biasing. Since for most humans vision is the dominant sense, visual attention is critically important for higher-order cognitive functions and related deficits are a core symptom of many neuropsychiatric and neurological disorders. Here, we summarize the importance and relative contributions of different neuromodulators and neurotransmitters to the neural mechanisms of top-down and bottom-up attentional control. We will not only review the roles of widely accepted neuromodulators, such as acetylcholine, dopamine and noradrenaline, but also the contributions of other modulatory substances. In doing so, we hope to shed some light on the current understanding of the role of neurochemistry in shaping neuron properties contributing to the allocation of attention in the visual field.

## Introduction

### Definition of Visual Attention

The term “visual attention” refers to cognitive processes that allow us to selectively process the vast amount of information we are confronted with every day. Since the capacities of the perceptual system are limited, focusing on a certain aspect of the visual field enables us to prioritize relevant information and ignore irrelevant information. Even though there are many definitions of attention, the following review will mainly be concerned with visuospatial selective attention.

### Bottom-Up and Top-Down Attention

This biasing of input is implemented through different types of attention. One of the most common distinctions is made between top-down and bottom-up attention (Corbetta and Shulman, 2002). Top-down refers to the voluntary guidance of attention by internal goals, whereas bottom-up attention refers to the involuntarily capture of attention by salient events in the environment. These terms describe if attention is allocated based on events in the sensory periphery (bottom-up) or from higher cortical areas (top-down). Both attention modes have been associated with distinct neural processes, but seem to be closely connected (Katsuki and Constantinidis, 2014). For example, it was found that top-down beta band influences causally increased bottom-up gamma-band influences from early visual areas (Richter et al., 2017). However, the apparant dichotomy of top-down and bottom-up attention has been challenged recently, since evidence suggests that there might be other factors controlling visual selection, such as reward-based history effects (Awh et al., 2012; Failing and Theeuwes, 2018). Together, these processes modulate neural activity within the visual system and thus shape how we perceive our environment. It has to be kept in mind though that visual attention and working memory processes are difficult to separate and highly interactive (Awh et al., 2006; Theeuwes et al., 2009).

### Focus and Motivation of Review

Visual attention is an important component of higher-level cognition, particularly in humans for whom vision is the dominant sense. Thus, deficits in visual attentional processing are a core symptom of many neurological and neuropsychiatric disorders. In recent years, there has been an ever-growing number of studies dedicated to the field of visual attention processing, especially to the important question of underlying mechanisms. This increasing interest is supported by recent advances in technology. Electrophysiological and neuroimaging methods have provided new insights into the human brain while it is engaged in cognitive work (He et al., 2011). Non-invasive stimulation methods, like transcranial magnetic stimulation (TMS) and transcranial direct current stimulation (tDCS), have been used to influence the excitability of cortical tissues and to investigate the cognitive functions of brain regions (Sparing and Mottaghy, 2008). In addition, magnetic resonance spectroscopy (MRS) measures the concentration of metabolites in different tissues and allows for the *in vivo* study of biochemical processes in the brain (Tognarelli et al., 2015). However, despite the growing interest in the topic and the development of new techniques, there are still many open questions concerning the mechanistic basis of visual attention (Moore and Zirnsak, 2017). Until now, studies targeting the neurophysiology or neurochemistry behind attentional processing have focused on top-down attentional processes. It can be assumed that bottom-up attention also modulates neural activity within the visual system, but this mechanism is comparably less well understood (Noudoost et al., 2010; Moore and Zirnsak, 2017; Thiele and Bellgrove, 2018). This is unfortunate, because several neuropsychiatric disorders have been linked to deficits in bottom-up attentional processing, such as schizophrenia (Javitt, 2009; Neuhaus et al., 2011) and ADHD (Schneidt et al., 2018). In contrast to earlier works, we want to give a more complete overview of the neurochemical influences on both aspects of visual attention. Furthermore, we aim to include neuromodulators and neurotransmitters that other reviews have so far neglected. In doing so, we hope to shed some light on aspects of visual attentional processing that have not yet been thoroughly investigated.

## Neural Basis of Attentional Processing

### Brain Regions Involved in (Top-Down and Bottom-Up) Visual Attention

Visual attention is by definition a selective process. It facilitates the processing of information based on their visual saliency or inherent goals, while irrelevant stimuli are filtered out. Neurophysiological and neuroimaging studies have provided valuable information about the brain regions involved in this filtering process. Generally, it is believed that the influence of attention increases along a cortical hierarchy, characterized by expanding size and complexity of receptive fields (Serences and Yantis, 2006). However, early attentional effects are already detectable at V1 (Katsuki and Constantinidis, 2014). From there, bottom-up processing of visual information ascends to higher cortical areas through two major pathways. The ventral pathway, comprising posterior visual areas (V1–V4), the inferior temporal cortex and the ventral prefrontal cortex, is assumed to deal with object- and feature-based visual processes. The dorsal pathway on the other hand is supposed to deal with spatial- and movement-related processes and includes V1, V2, and V3, middle temporal regions, the posterior parietal cortex and the dorsolateral prefrontal cortex (Mishkin and Ungerleider, 1982; Ungerleider and Haxby, 1994; Katsuki and Constantinidis, 2014). By contrast, the top-down selection of visual information is supported by prefrontal and parietal areas and exerts its influence primarily through feedback connections. Particularly brain regions associated with oculomotor or gaze function, such as the frontal eye fields (FEF) and the intraparietal sulcus (IPS), seem to be involved in voluntary attentional control (Noudoost et al., 2010; Meehan et al., 2017; Thiele and Bellgrove, 2018).

### Computational Theories of Visual Attention

Computational theories of attention provide a closer look at the neural mechanisms by which relevant information are filtered from the environment (Koch and Ullman, 1985; Itti and Koch, 2001). They propose that bottom-up attention is implemented through a mechanism, which directs attention to the most salient stimulus in the visual field. Therefore, it is assumed that the basic visual features supporting the selection of a certain stimulus (e.g., orientation, luminance or color) are represented in separate feature maps, which are then combined into a topographically oriented global saliency map. Based on a winner-take-all-mechanism attention increases neuronal activity for the winning neuron population and suppresses all competing populations. Thus, attention is directed to the spot on the salience map which shows the most activation (Katsuki and Constantinidis, 2014). However, attention allocation is not only determined by stimulus salience but also by internal goals. This led to the postulation of a priority map, which is supposed to integrate top-down and bottom-up factors (Serences and Yantis, 2006; Bisley and Goldberg, 2010). The idea of a priority map simultaneously modulated by both processes is supported by functional magnetic resonance imaging (fMRI) research showing that bottom-up and top-down attention increase activation in an overlapping frontoparietal brain network (Corbetta and Shulman, 2002; Katsuki and Constantinidis, 2012, 2014).

### Attentional Effects on Neuronal Activity

On the neural level, attention modulates the acitivity of neurons or neuron populations representing an attended location or feature by increasing their signal-to-noise-ratio (SNR; Noudoost et al., 2010; Paneri and Gregoriou, 2017; Sapountzis and Gregoriou, 2018). One way in which attention strengthens the selected signals is by modulation of the neuron’s firing rate. Another important effect of attention is a decrease in the correlated variability of visual neurons, which might be an even more significant factor with respect to general reductions in SNR than the modulation of firing rates (Cohen and Maunsell, 2009; Mitchell et al., 2009; Deco and Hugues, 2012; Paneri and Gregoriou, 2017). Additionally, attention might lead to frequency specific modulations of local oscillatory activity at different stages of visual processing (Gregoriou et al., 2015). These modulations include rhythmic synchronizations in the gamma band range (30–60 Hz) which might result in a more effective processing (Murthy and Fetz, 1994; Fries, 2005, 2009; Deco and Thiele, 2009; van Es and Schoffelen, 2019). Support for this assumption comes from neurophysiological studies that demonstrate long-range gamma coupling between visual neurons processing an attended stimulus (Gregoriou et al., 2009; Bosman et al., 2012; Grothe et al., 2012). Thus, competition between representations during attentional selection might be resolved by modulations of firing rate and inter-neuronal variability, but also by coordinated oscillatory activity (Paneri and Gregoriou, 2017). How these changes are brought about is still not completely clear. One option would be the involvement of neuromodulators.

## Neuromodulation

### General Information

Neuromodulation describes the alteration of neuronal and synaptic properties by neurons or substances released by neurons (Katz and Calin-Jageman, 2008). A wide variety of substances, including neurotransmitters, biogenic amines and neuropeptides can act as neuromodulators (Noudoost and Moore, 2012; Nadim and Bucher, 2014; Moore and Zirnsak, 2017). While there is no exact definition of the term “neuromodulator,” Picciotto et al. (2012) describe them as “any kind of neurotranmission that is not directly excitatory […] or inhibitory […]” (p.1, refering to Siggins, 1979; Ito and Schuman, 2008). The human neuromodulatory system includes noradrenergic, serotonergic, dopaminergic and cholinergic projections, which are thought to provide a foundation for many higher cognitive functions, such as attention, decision-making, emotion and goal-directed behavior (Avery and Krichmar, 2017). Neuromodulatory actions are generally mediated by G-protein-coupled receptors and affect ion channels or other membrane proteins. Thereby, neuromodulators can change intrinsic firing properties and alter the synaptic strength of neurons, as well as modify short-term and long-term plasticity (Gu, 2003; Sakurai and Katz, 2009; Picciotto et al., 2012; Nadim and Bucher, 2014). It has to be kept in mind though, that because of the complexity of neuromodulatory effects, the non-linear dynamics of neuromodulators and the simultaneous synaptic presence of several neuromodulators, the consequences for specific cortical processes, like visual attention, are difficult to comprehend (Nadim and Bucher, 2014).

### Neuromodulation of Visual Attention

Shifts in behavioral states (from sleep to wakefulness or from inattentive to vigilant) are connected to changes in global brain activity. These changes are at least partly controlled by neuromodulators (Lee and Dan, 2012). However, since attentional processes, such as top-down attention, were considered to be highly specific, whereas the actions and projections of most neuromodulators were not, their involvement in regulating selective attention remained controversal (Mesulam et al., 1986; Sarter et al., 2009). Only recently, it has been assumed that the global mechanisms governing cortical states may also operate on a local scale, for example during visual selective attention (Harris and Thiele, 2012; Rabinowitz et al., 2015; Engel et al., 2016). This assumption was supported by studies demonstrating that the same neuromodulators known to act on a brain-wide scale also modulate attentional effects (Zaborszky and Duque, 2003; Herrero et al., 2008; Harris and Thiele, 2012; Noudoost and Moore, 2012). For example, the neurotransmitter acetylcholine (ACh) has been implicated in vigilance and attention, as well as in the regulation of global brain states (Lee et al., 2005). Applying ACh to cells in primate V1, as well as enhancing ACh activity with cholinesterase inhibitors (donezepil) in humans seems to evoke neuronal effects similar to those of attention (Roberts et al., 2007; Gratton et al., 2017). Furthermore, attention can stabilize network states and decrease interneuronal correlation (Deco and Hugues, 2012). Interestingly, a decrease in neural variability is also found when stimulating the basal forebrain in rats, assumed to be associated with a local activation of ACh receptors (Goard and Dan, 2009). These and other similarities between attention and cholinergic activation have led to the suggestion that the neuromodulator ACh might be involved in attentional processing (Lee and Dan, 2012).

## Neuromodulators

Neuromodulators most often implicated in attentional processes include ACh and dopamine (Noudoost and Moore, 2011; Froemke, 2015; Thiele and Bellgrove, 2018). However, in the following section we will also review other important neuromodulators and their influence on voluntary and involuntary visual attention.

### Acetylcholine

#### General

ACh is an important neurotransmitter at the neuromuscular junction. In the brain its actions are primarily neuromodulatory (Picciotto et al., 2012). While ACh is synthesized in several different nuclei, its most important source is the basal forebrain. From there cholinergic projections innervate neocortex, hippocampus and amygdala (Mesulam et al., 1983; Woolf, 1991; Thiele, 2013). ACh receptors are typically categorized based on their binding capacity for muscarine and nicotine and thus divided into metabotropic muscarinic receptors (mAchRs) and ionotropic nicotinic receptors (nAChRs). Both receptor types show a widespread distribution within the nervous system (Gotti et al., 2006; Thiele, 2013) and there is evidence that they are both involved in attentional control (Noudoost and Moore, 2011). ACh has been implicated in attentional processes for quite a long time (Bartus et al., 1982; Zaborszky et al., 2002; Weinberger, 2007; Sarter et al., 2009; Newman et al., 2012; Carcea and Froemke, 2013; Ballinger et al., 2016). It is assumed to enhance attentional effects in the visual cortex by improving neuronal tuning (Sajedin et al., 2019) and modulating the spatial properties of receptive fields (Roberts et al., 2007; Gratton et al., 2017). Additionally, ACh seems to alter the covariance structure of cortical networks and to increase their coding capacity (Minces et al., 2017; Thiele and Bellgrove, 2018). Whereas earlier studies assumed that the cholinergic system would lack spatial and functional specificity (Mesulam et al., 1986; Sarter et al., 2009), recent findings argue that the spatial specificity of cholinergic projections is better than previously believed (Thiele, 2013; Wu et al., 2014; Unal et al., 2015; Zaborszky et al., 2015, 2018; Jiang et al., 2016; Gielow and Zaborszky, 2017). In this context, it was demonstrated that ACh not only shows slow tonic activity (Manns et al., 2000; Lee et al., 2005), but also precise phasic activities, associated with cue detection performance in attention tasks (Parikh and Sarter, 2008; Sarter et al., 2009; Gritton et al., 2016).

#### Effects of ACh Modulation on Visual Attention

Most earlier studies examined rodents and non-human primates to establish that attention allocation leads to changes in neuronal activity throughout the visual system. In monkeys, the application of ACh augmented their responses to attended stimuli, while the application of an mAchR antagonist (scopolamine) reduced the modulatory effect of attention (Herrero et al., 2008). In humans, the acute cognitive effects of nAChR agonist nicotine on attention but also on sensory processing and memory are well established (Heishman et al., 2010; Hahn, 2015; Hahn et al., 2020). Evidence for ACh involvement in attentional modulation comes from a variety of studies using different methodologies. Using magnetic resonance spectroscopy, Lindner et al. (2017) measured choline as an indirect marker of ACh availability in the brain. By measuring choline levels in the parietal cortex during a visuospatial attention task, they found direct evidence for the involvement of cholinergic systems in human visual attention. In a double-blind, placebo-controlled crossover fMRI study Ricciardi et al. (2013) assessed cholinergic effects on brain connectivity and blood-oxygen-level-dependent (BOLD) signal variability during a visual selective attention task (matching task with superimposed faces and houses). They found that cholinergic enhancement by physostigmine (acetylcholinesterase inhibitor) improved task performance as well as reduced BOLD signal variability and functional connectivity between ventral visual processing areas and task-relevant regions. These findings were concluded to be in line with the assumption that cholinergic modulation enhances neural efficiency reflected by less BOLD signal variability in visual regions (Ricciardi et al., 2013). Research targeting the genetics of cholinergic influence on visual attention investigated the choline transporter (CHT), which is important for the synthesis and release of ACh (Sarter et al., 2016). The authors found that genetically reduced CHT activity leads to poorer top-down attentional control and more attentional distraction, whereas elevated levels of CHT may increase resistance to distraction. Moreover, it was found that ACh modulates neural oscillations during attentional processing (Bauer et al., 2012; Howe et al., 2017). In rats, for example, detection of visual cues was associated with phasic ACh release and increases in high and low frequency power. In addition, nAChR and mAChR antagonists (mecamylamine, telencepine) reduced power in the high gamma frequency, most likely related to cue-triggered reorienting effects (Howe et al., 2017). In humans, the cholinergic agonist physostigmine has been shown to enhance visual attention effects on low-level alpha and beta oscillations (Bauer et al., 2012). After establishing that ACh plays a major role in modulating attentional processes, the following section will try to dissociate the contributions of nicotinic and muscarinic receptor types to this modulatory effect.

#### Dissociable Effects of nAChR and mAChR

Both, the nACh and the mACh receptor allow ACh to modulate electrical activity of cortical neurons and to affect intracellular signaling (Thiele, 2013; Yakel, 2013). In addition, both have been implicated in higher cognition, particularly in attention and memory (Friedman, 2004; Green et al., 2005; Sarter and Parikh, 2005; Ellis et al., 2006).

Concerning nicotinic involvement in visual attention, it was found that in human participants nicotine (administered as NICORETTE polacrilex gum) improved attentional reorienting in a visuospatial cued detection task by a reduction of neural activity in parietal brain regions of non-smoking participants, measured by fMRI (Thiel et al., 2005). In another fMRI study, Hahn et al. (2009) found that nicotine (administered as transdermal patch) reduced reaction times specifically for selective attention tasks (compared with a divided attention task), while also reducing BOLD activity in several cortical and subcortical regions and increasing deactivations within the default mode network. Thus, the effects of nicotine on attention seem to be task-dependent with a stronger impact on simple detection tasks, probably induced by an enhanced functional efficiency and a downregulation of task-independent default activity (Hahn et al., 2009). Furthermore, applying a nicotine antagonist (Mecamylamine) led to disturbances in visual attention and fine motor tasks, which could be reversed by the administration of transdermal nicotine (Alvarez-Jimenez et al., 2018). Using a CombiTVA paradigm, Vangkilde et al. (2011) showed that nicotine (administered as Nicotinelle polacrilex gum) lowers the perceptual threshold, speeding up short-term memory transfer, but slowing down subsequent information processing and weakening top-down selective attention. In addition, it was demonstrated that normal variation in a nicotinic receptor gene modulated visuospatial attention, but dopamine gene variability did not (Greenwood et al., 2005; Parasuraman et al., 2005). However, low doses of an nAChR antagonist (mecamylamine) did not seem to affect spatial selective attention, stimulus detection or visual information processing (Yuille et al., 2017).

Besides, there is also evidence of muscarinic involvement in attentional modulation. In rodents, mACh transmission influences visual processing in V1 (Kang et al., 2014), improving neural sensitivity and increasing long-term responsiveness (Gu, 2003; Kang and Vaucher, 2009; Kang et al., 2014). Using iontophoretic pharmacological analysis and single-cell recordings in V1 neurons of macaque monkeys performing a top-down spatial attention task, it was found that low doses of ACh enhanced attentional modulation in V1 neurons, while applying scopolamine (mACh antagonist) reduced attentional modulation and mecamylamine (nACh antagonist) had no effect (Herrero et al., 2008). In humans, Laube et al. (2017) found that the mAChR antagonist scopolamine modulated top-down attentional control processes during a contingent capture task by reducing an EEG component associated with active suppression of irrelevant distractors. Additionally, Ellis et al. (2006) showed that muscarinic (scopolamine), but not nicotinic (mecamylamine) blockade reduced performance in sustained attention measured by digit vigilance and rapid visual information processing tasks.

However, there is also the idea that nicotinic and muscarinic influences might have differential effects, but work synergistically to shape attentional processing (Hasselmo, 2005; Ellis et al., 2006; Greenwood et al., 2009; Dasilva et al., 2019). For example, it was proposed that ACh would enhance thalamocortical transmissions through nicotinic receptors, boosting bottom-up inputs. Top-down attentional effects, on the other hand, would be reduced by muscarinic inhibition (Hasselmo, 2005; Greenwood et al., 2009). These differences in cholinergic actions across brain regions might be supported by the different expression profiles of nicotinic and muscarinic receptors from primary sensory to higher cortical areas (Galvin et al., 2018). For example, it was found that in macaque early visual areas (V1) mACh receptors were predominantly expressed by gamma-aminobutric acid (GABA) ergic cells while in higher regions they also occured on pyramidal cells. In V1 nicotinic β2 receptors are expressed presynaptically on thalamocortical cells (Disney et al., 2007; Galvin et al., 2018), whereas in higher cortical regions, nACh receptors are expressed on glutamatergic inputs to layer V cells as well as on layer V interneurons and pyramidal neurons (Poorthuis et al., 2013). Furthermore, mACh and nACh receptors showed cell-type specific activity by promoting attentional control signals through muscarinic receptors in broad spiking cells and through muscarinic and nicotinic cells in narrow spiking cells (Dasilva et al., 2019).

#### ACh Effects on Bottom-Up and Top-Down Visual Attention

It is believed that ACh has a greater effect on top-down attentional modulation than on bottom-up cue detection (Rokem et al., 2010; Galvin et al., 2018). The cholinesterase inhibitor donezepil selectively improved performance in long SOA trials of a spatial cueing task, thus, enhancing voluntary attention, without affecting involuntary attention. Therefore, it seems likely that top-down and bottom-up attention rely on “different neurochemical mechanisms” (Rokem et al., 2010). A recent study used a contingent capture paradigm and the muscarinic receptor antagonist scopolamine to test the involvement of muscarinic receptor activation in the modulation of top-down attentional control. While scopolamine did not affect early visual feature-enhancement or attentional capture by task-relevant features, it did reduce a measure associated with active suppression of distractors, pointing toward a cholinergic modulation of top-down attentional control (Laube et al., 2017). Other studies found that an increase in ACh seems to augment visual attention through an increase of activity in visual regions and a reduced top-down biasing of sensory information (Bentley et al., 2004; Vangkilde et al., 2011). Thus, apart from the influence of ACh on voluntary attentional control, there is also some evidence of cholinergic influence on bottom-up attention. Studies examining Parkinson’s disease patients have demonstrated the importance of thalamic cholinergic integrity for bottom-up attentional mechanisms (Kim K. et al., 2017). Animal and human studies on bottom-up attention show that attentional orienting to peripheral cues is supported by cholinergic activity in the basal ganglia (Voytko et al., 1994; Witte et al., 1997; Murphy and Klein, 1998). A study with non-human primates indicates that covert attentional orienting might rely on muscarinic cholinergic activity in the intraparietal cortex (Davidson and Marrocco, 2000). Furthermore, it was found that ACh enhances the response to external sensory information while reducing background noise (Hasselmo and McGaughy, 2004; Hasselmo, 2005). This sharpening of visuospatial representations might favor bottom-up attentional processes. Interestingly, it seems that cholinergic effects on top-down and bottom-up attention are not incompatible. As already mentioned, top-down and bottom-up effects of ACh might rely on the interplay between muscarinic (top-down) and nicotinic (bottom-up) ACh receptors (Hasselmo, 2005; Greenwood et al., 2009). In addition, there is evidence that the two attentional processes might be dissociated by temporal ACh release properties. Whereas bottom-up attention involves phasic ACh release in interaction with thalamocortical circuits (Gritton et al., 2016; Sarter and Lustig, 2019), top-down attention is supported by longer-timescale cholinergic modulation of right fronto-parietal circuity (Paolone et al., 2010; St. Peters et al., 2011; Sarter et al., 2016; Sarter and Lustig, 2019). This is in line with the assumption that cholinergic activity establishes biases based on the interaction of top-down and bottom-up processes that synergistically modulate stimulus representation. Furey et al. (2008) for example demonstrated that ACh alters the salience of the attended stimulus and its competing stimuli, influencing top-down as well as bottom-up processing. The finding that one neuromodulator might influence voluntary as well as involuntary attentional processes is consistent with the idea that both processes are closely connected (Katsuki and Constantinidis, 2014). In this context, Kanamaru and Aihara (2019) proposed a network in which top-down ACh can modulate and support bottom-up inputs by increasing the firing rate and changing the response function of visual neurons, further emphasizing the idea that ACh enhances the processing of attended stimuli (Gratton et al., 2017).

#### Summary

ACh is the neuromodulator that has been most often implicated in attention. It also plays an important role in the processing of visual attention (Ricciardi et al., 2013; Sarter et al., 2016; Lindner et al., 2017; Galvin et al., 2018) and might be involved in the modulation of related brain oscillations (Bauer et al., 2012; Howe et al., 2017). Both, nicotinic and muscarinic receptor types, influence attentional processing (Ellis et al., 2006; Thiele, 2013; Laube et al., 2017; Alvarez-Jimenez et al., 2018; Hahn et al., 2020). However, the specific roles of nicotinic and muscarinic activation are not yet fully understood. It is hypothesized that they have differential effects on attentional processes, but work synergistically (Hasselmo, 2005; Greenwood et al., 2009) by implementing the influences generated by top-down and bottom-up signals (Furey et al., 2008; Sarter et al., 2016; Gratton et al., 2017; Kanamaru and Aihara, 2019).

### Dopamine

#### General

Dopamine (DA) is a monoamine neurotransmitter, synthesized in the ventral tegmental area and the substantia nigra of the brain (Juárez Olguín et al., 2016). Unlike ACh and other substances, DA does not act as a fast ionotropic neurotransmitter, but seems to mainly influence other receptor channels (Yang and Seamans, 1996; Seamans and Yang, 2004; Vitay and Hamker, 2007). There exist five subtypes of DA receptors: D1, D2, D3, D4, and D5, which are divided into two subclasses, D1-like receptors (D1 and D5) and D2-like receptors (D2, D3, D4; Ayano, 2016). Dopaminergic neurons are widely distributed, in the central nervous system and in the periphery, where they are necessary for the maintenance of many physiological processes. In the brain, the dopaminergic systems play an important role in the neuromodulation of cognitive functions, motor control, motivation, reward, attention and learning. However, DA seems to be especially relevant when it comes to cognitive control and executive function (Floresco and Magyar, 2006; Cools, 2016). Thus, unbalanced activity or dopaminergic dysfunction has been associated to the pathophysiology of several psychiatric and neurodegenerative disesases, such as schizophrenia, mood disorders, obsessive compulsive behavior, autism spectrum disorders, attention deficit-hyperactivity disorder, Tourette’s syncrome, substance dependency and Parkinson’s disease (Ayano, 2016).

#### Effects of DA Modulation on Visual Attention

A dopaminergic role in reward anticipation, reward prediction and learning is already well established (Schultz, 1998; Schott et al., 2008; Steinberg et al., 2013; Thiele and Bellgrove, 2018). Therefore, it is not surprising that the effect of DA on visual attention has been connected to reward processing. For example, it was suggested that visual processing is influenced by the amount of reward associated to a stimulus and that this association is modulated by dopaminergic signals (for review see: Vitay and Hamker, 2007). According to the “incentive salience” hypothesis of Berridge and Robinson (1998) DA influences the visual representations of reward-associated stimuli and makes them more salient. In line with this finding, it was shown that DA is involved in reward-driven modulations of attentional control. For example, incentive reward enhances activity in a network closely associated with DA release and attention (Engelmann et al., 2009). Consistently, it was found that DA improves the signal-to-noise-ratio of neurons at downstream processing sites (Kroener et al., 2009; Pessoa, 2015; Vander Weele et al., 2018), indicating that dopaminergic modulation provides a mechanism by which reward “sharpens” attentional control, thereby enhancing the processing efficiency in cortical and subcortical regions (Failing and Theeuwes, 2017). However, attention and reward are difficult to dissociate (Maunsell, 2004; Peck et al., 2009; Noudoost and Moore, 2012) and there is also evidence that DA signals salient stimuli in absence of reward (Horvitz, 2000).

Possibly independent from its role in reward, DA seems to be central for the processing of attention (Nieoullon, 2002). Prefrontal cortex (PFC) projections of DA-producing neurons in the ventral tegmental area and in the substantia nigra seem to be closely related to several aspects of attention, from inhibitory control to sustained and selective attention (Briand et al., 2007; Noudoost and Moore, 2011; Chandler et al., 2014; Clark and Noudoost, 2014; Ranganath and Jacob, 2016; Shalev et al., 2019). Moreover, it is assumed that the PFC represents the main source of top-down signals biasing attentional selection in early visual areas (Paneri and Gregoriou, 2017; Mueller et al., 2020). Interestingly, optimal PFC functioning seems to rely on moderate D1 receptor stimulation, as well as on the stimulation of noradrenergic α_2A_ receptors (Arnsten and Pliszka, 2011).

However, the PFC is not the only brain region associated with dopaminergic modulation of attention. DA is also an important determinant of basal ganglia (BG) function—a brain region that is known for its involvement in eye movement generation and attentional control (Hikosaka et al., 2000). Striatal DA has been implicated in reward processing (Berridge and Robinson, 1998; Haber, 2011; Deserno et al., 2016; Katthagen et al., 2020), voluntary motor behavior (Albin et al., 1989; Joshua et al., 2009; Klaus et al., 2019), learning and motivation (Berridge and Robinson, 1998; Waelti et al., 2001; Graybiel, 2008) as well as in the involuntary attention orienting toward reward-related cues (Anderson et al., 2016). For example, Cameron et al. (2018) orally administered tolcapone capsules (Catechyl-O-Methyltransferase (COMT) inhibitor) and bromocriptine capsules (D2 agonist) to human participants performing a visual attention task, that required switching between pro- and anti-saccade responses. Greater prefrontal DA tone led to a reduced performance in the visual task. The authors concluded that DA might facilitate inhibitory control, which could have resulted in an excessive focusing of attention and might have been sub-optimal for the task at hand.

Dopaminergic activity has also been associated with several attention networks: the dorsal attention network (DAN, externally controlled attention; Corbetta and Shulman, 2002), the default mode network (DMN, internal cognitive processes; Raichle et al., 2001; Raichle, 2015) and the fronto-parietal control network, which is assumed to mediate the allocation of attentional resources between the two other networks (FCPN; Spreng et al., 2010). It was found that blocking DA reuptake increases DAN activation during visual attention tasks (Müller et al., 2005; Tomasi et al., 2011) and alters DMN activation (Tomasi et al., 2011). Additionally, Dang et al. (2012) demonstrated that DA influenced the interaction between DAN, DMN and FPCN.

Further evidence for the role of DA in attention comes from studies focusing on inattention. As it was shown, inattention can be attributed to disruptions of DA activity. Gorgoraptis et al. (2012), for example, demonstrated that a dopaminergic agonist (transdermal rotigotine) had beneficial effects on visual search and selective attention in patients with hemispatial neglect following stroke. Some stimulant medications used in the treatment of inattention, like methylphenidate and amphetamine, enhance dopaminergic activity by targeting DA transporters (DAT; Seeman and Madras, 1998; Wang et al., 2013; Robison et al., 2017). In line with this mode of action, Tomasi et al. (2009) found that lower DAT (higher DA activity) facilitated visual attention by modulating brain deactivations in default mode regions, such as the precuneus.

On a side note, DA also seems to have a direct influence on retinal vision by tuning visual processing through cellular and circuit-level mechanisms within the retina (Witkovsky, 2004; Roy and Field, 2019). For instance, DA signaling has been shown to impact spatial and temporal receptive fields of retinal neurons (Chaffiol et al., 2017; Mazade et al., 2019).

#### DA Effects on Bottom-Up and Top-Down Visual Attention

Dopaminergic activity in the PFC is important for a variety of cognitive processes, such as working memory, motivation and action planning (Ayano, 2016). At the same time, the PFC is assumed to be essential for the top-down regulation of attention through long-range projections upon visual areas (Baluch and Itti, 2011; Noudoost and Moore, 2011; Paneri and Gregoriou, 2017). Recently, it was demonstrated that DA modulation in the PFC operates through phasic activation of dopaminergic neurons that modify PFC activity and gamma oscillations (Lohani et al., 2019). Some evidence for the role of DA in top-down attentional control comes from Prinzmetal et al. (2010). The authors investigated the effect of D2 receptor agonist bromocriptine in a visual cueing task targeting spatial attention. Bromocriptine enhanced the effect of spatial cueing for long Stimulus-Onset-Asynchrony (SOA), but not for short SOA trials. Thus, dopaminergic modulation seems to affect voluntary but not involuntary spatial attention. One region of the PFC, the frontal eye field (FEF), is believed to be particularly important in attentional control and seems to be modulated by dopaminergic activity (Noudoost and Moore, 2011, 2012; Chandler et al., 2014; Mueller et al., 2020). Noudoost and Moore (2011) examined the effect of D1 receptor blockade on FEF activity in macaques. They used microinjections to applicate D1 receptor antagonists (SCH23390) into sites within the FEFs while simultaneously recording activity from matching sites in V4. The authors found that manipulating FEF activity increased target selection, but also magnitude, selectivity and reliability of V4 responses. These effects were concluded to be similar to those of covert attention in absence of a behavioral task (Noudoost and Moore, 2011). In another experiment, the authors used a D2 receptor agonist (quinpirole), which also led to an increase in target selection. However, only D1 receptor manipulation affected V4 responses (Chudasama and Robbins, 2004; Noudoost and Moore, 2011; Yousif et al., 2016). To explain these discrepant results, it was suggested that D1 receptors are more involved in generating top-down attention signals than D2 receptors. Thiele and Bellgrove (2018) concluded that this functional dissociation might be explained by the differing amounts of D1 and D2 receptors in supragranular and infragranular layers of the FEF (with D1 receptors being expressed in both layers and D2 receptors being expressed only in infragranular layers; Lidow et al., 1991). While dopaminergic input derived from the substantia nigra preferentially terminates in supragranular layers and could be more involved in increasing attentional feedback signals, dopaminergic input derived from the ventral tegmental area preferentially terminates in infragranular layers and could be more involved in reward and error processing.

However, the FEFs have not only been associated with top-down control but also with bottom-up processing. Based on visual search experiments in non-human primates it is assumed that the FEFs might contain the neural correlate of a salience map or be part of a distributed network representing visual salience (Thompson and Bichot, 2005). As such, the FEFs would process top-down as well as bottom-up factors (Thompson and Bichot, 2005; Katsuki and Constantinidis, 2012, 2014; Joiner et al., 2017). In line with this assumption, there is some evidence of dopaminergic involvement in bottom-up visual attention. Lundwall et al. (2012), for example, demonstrated that exogenous orienting was associated with dopaminergic markers on COMT and DAT1. Anderson et al. (2016) explored the contribution of DA to value-based attention in a positron-emission-tomography (PET) study. They found that DA signaling in the striatum was linked to involuntary attention orienting—particularly to the exogenous attraction of attention by reward cues. Enhanced levels of DA were associated with reward-related attentional capture, while suppression of DA release was associated with the ability to ignore reward-related stimuli. Contrary to these results, Rokem et al. (2012) reported no effect of DAT1 genotype or D2 receptor agonist bromocriptine on involuntary attention.

#### Summary

Dopaminergic neurons contribute to a variety of cognitive functions and disruptions of dopaminergic activity are associated with several psychiatric and neurodegenerative dieseases. With regard to visual attention, DA seems to exert its influence primarily through dopaminergic activity in the PFC/FEF and related brain networks (DAN). These regions have been shown to modulate attention via top-down projections upon the visual cortex (Noudoost and Moore, 2011). A recurring finding is that the effect of DA is dependent on receptor type and localization. D1 and D2 receptors have been found to be differentially expressed within different layers of the primate frontal cortex and while DA acts on both receptor types, its main actions related to top-down attention occur at the D1 receptor, which is found in superficial and deeper layers of the PFC (Lidow et al., 1991; Arnsten and Pliszka, 2011; Soltani et al., 2013; Mueller et al., 2020).

### Noradrenaline

#### General

The Locus Coeruleus (LC) is known as the main source of noradrenaline (NA) in the brain. In this region, NA is synthesized directly from DA by the dopamine-β-hydroxylase (Loizou, 1969; Bari et al., 2020). There exist several subtypes of adrenergic receptors, most often classified in α and β families, which can again be subdivided into α_1_ and α_2_ or β_1_, β_2_ and β_3_ receptor categories (Strosberg, 1993; Xing et al., 2016). NA has the highest affinity for α_2_-receptors (Arnsten, 2000) and the α_2A_ subtype is most prevalent within the PFC. NA seems to be broadly released in the brain (Levitt and Moore, 1978), in tonic and phasic firing modes. These two modes have been associated with arousal and wakefulness on the one hand and novelty and behaviorally driven exploration on the other hand (Vankov et al., 1995; Berridge and Waterhouse, 2003; O’Donnell et al., 2012; Aston-Jones and Waterhouse, 2016; Thiele and Bellgrove, 2018).

#### Effects of NA on Top-Down and Bottom-Up Visual Attention

DA and NA show overlapping effects on learning, brain state and reward processing (Ranjbar-Slamloo and Fazlali, 2020). They are also implicated in modulating attention (Clark et al., 1989; Ward and Brown, 1996; Coull et al., 2001; De Martino et al., 2008; Borodovitsyna et al., 2017). In addition, both catecholamines are assumed to synergistically modulate PFC functioning (Xing et al., 2016), which might imply that they are both involved in top-down processing of attention (Arnsten and Pliszka, 2011). However, Shalev et al. (2019) demonstrated that the effects of DA and NA on visual attention can be dissociated. They found that attentional selection was associated with the COMT genotype, while sustained attention was linked to the dopamine-beta-hydroxylase (DBH) genotype. NA also shares similarities with ACh concerning its role in attentional processing (Thiele and Bellgrove, 2018). For example, using a Bayesian statistical framework, it was suggested that NA and ACh control selective attention, flexible information processing and learning by forming an interactive, partly synergistic, partly antagonistic relationship in the representation of expected (ACh) and unexpected (NA) uncertainty (Yu and Dayan, 2005).

In general, noradrenergic activity has been found to influence attentional orienting (Coull et al., 2001; Sara and Bouret, 2012), disengagement of attention (Clark et al., 1989) and attentional shifting (Snyder et al., 2012). Specifically, NA seems to be involved in salience detection (Foote et al., 1980; Aston-Jones and Bloom, 1981; Servan-Schreiber et al., 1990; Berridge and Waterhouse, 2003; De Martino et al., 2008; Markovic et al., 2014; Mather et al., 2016; Gelbard-Sagiv et al., 2018). In addition, noradrenergic signaling is supposed to enhance the ability to discriminate between relevant and irrelevant information (Aston-Jones and Cohen, 2005) and thus bias attentional tuning (Ehlers and Todd, 2017). For example, Gelbard-Sagiv et al. (2018) manipulated noradrenergic activity using clonidine (an α2 agonist, should reduce NA activity and task performance) and reboxetine (selective NA reuptake inhibitor, should increase NA signaling) and examined their effect on visual perception, EEG and fMRI responses. They found that visual detection and stimulus discrimination were affected by NA manipulations as expected, while decision bias and sustained attention were not. Moreover, they found that the reduction of NA signaling affected ERP components and brain regions associated with visual awareness. These results indicate that NA influences primarily the later stages of perceptual processing and not attentional function *per se* (Gelbard-Sagiv et al., 2018; see also Nieuwenhuis et al., 2007). However, in the study by Gelbard-Sagiv et al. (2018) reboxetine did show a trend toward an increased performance in the sustained attention task. If so, the authors conclued, bottom-up attentional processes might be more in line with noradrenergic influences on sensory processing. Underlining the role of NA in stimulus-associated processing, several studies found that NA as well as ACh suppressed top-down information in favor of bottom-up sensory information (Hasselmo et al., 1996; Kimura et al., 1999; Kobayashi et al., 2000; Yu and Dayan, 2005). Contrary to this, Dockree et al. (2017) demonstrated that methylphenidate, a mixed DA and NA reuptake inhibitor, improved sustained attention by acting on top-down attentional control with no effect on stimulus-driven, sensory processes.

Interestingly, as with dopaminergic D1 receptor stimulation, it was demonstrated that insufficient as well as extreme catecholaminergic activity can have negative effects on attention performance (Berridge and Waterhouse, 2003; Aston-Jones and Cohen, 2005; Arnsten and Pliszka, 2011; Shalev et al., 2019). While moderate levels of NA have been shown to improve PFC function by acting on α_2_ receptors, high levels of NA might decrease PFC function through α_1_ or β receptors (Arnsten, 2000; Lapiz and Morilak, 2006; Arnsten and Pliszka, 2011). The opposing effects of high and low NA concentration on distinct adrenoceptors with different affinities could also be the reason for the discrepant results concerning bottom-up and top-down attentional control. This conflict might be resolved by assuming that α_2_ receptors in the PFC regulate top-down attention (Coull et al., 2001; Robbins and Arnsten, 2009; Mather et al., 2016) and α_1_ receptors in the sensory cortex modulate bottom-up processes (Thiele and Bellgrove, 2018).

#### Summary

There is evidence that NA primarily influences visual perception (Gelbard-Sagiv et al., 2018) and salience detection (Markovic et al., 2014; Mather et al., 2016). However, the role of moderate noradrenergic levels in enhancing PFC functions seem to imply an additional effect on top-down attention, working memory, decision-making and emotional regulation (Berridge and Waterhouse, 2003; Arnsten, 2009; Ehlers and Todd, 2017). The heterogeneous role of NA in biasing attentional processes could be explained by taking into account the different localizations, affinities and effects of adrenergic receptors. While top-down effects might depend on moderate levels of NA enhancing PFC function, acting on α2 receptors; bottom-up effects might be associated with higher levels of NA, decreasing PFC function by acting on α1 or β receptors (Arnsten and Pliszka, 2011; Thiele and Bellgrove, 2018).

### Serotonin

#### General

Serotonin (5-hydroxytryptamine, 5-HT) is synthesized from L-tryptophan and released by serotonergic neurons in the Raphe nuclei of the brainstem (Mohammad-Zadeh et al., 2008; Walker and Tadi, 2020). There exist at least fourteen 5-HT receptor subtypes (Hoyer et al., 1994), which regulate distinct physiological processes (Hoyer and Martin, 1997; Stiedl et al., 2015). The most prevalent and extensively researched receptor types are the 5-HT1A and the 5-HT2A receptors (Carhart-Harris and Nutt, 2017). 5HT is mostly found outside the central nervous system, where it regulates biological processes associated with cardiovascular, pulmonary or gastrointestinal functions (Berger et al., 2009). However, 5-HT is also important within the central nervous system and seems to regulate almost all brain functions. Accordingly, it has been implicated in the pathogenesis of several psychiatric and neurological disorders (Berger et al., 2009).

#### Effects of 5-HT Modulation on Top-Down and Bottom-Up Visual Attention

5-HT receptors, especially 5-HT2A receptors, are highly expressed in the PFC (Puig and Gulledge, 2011). Consequently, 5-HT has been implicated in executive functions, emotion and motivation, learning and memory (Meneses and Liy-Salmeron, 2012). While serotonin activity seems to play an especially important role in prepotent response inhibition and impulsivity with less impact on attentional processing (Robbins, 2002; Brown et al., 2012; Worbe et al., 2014), it also seems to have some influence on attentional processing (Carter et al., 2005; Scholes et al., 2007; Wingen et al., 2008; Enge et al., 2014; Li et al., 2018). Carter et al. (2005) found that treatment with psyilocybin, a mixed 5-HT1A and 5-HT2A agonist, impaired the ability to suppress distractors and subsequently reduced attentional performance. Since a pretreatment with the 5-HT2A agonist ketanserin did not modulate the effect of psilocybin, the authors assumed that attentional processes primarily rely on 5-HT1A receptors. Even so, genetical manipulation of neurons expressing 5-HT2C receptors in the hippocampus exclusively modulated performance in a visual attention task (Li et al., 2018). Application of a selective serotonin reuptake inhibitor (escitalopram) led to a reduced activation in areas concerned with sustained attention (frontal regions, the thalamus and nucleus caudate, measured by fMRI; Wingen et al., 2008). Behaviorally, participants reported decreased alertness, but there was no effect of escitalopram on the performance of a sustained attention task (Mackworth Clock Test). Concerning attentional selection, an investigation of genetic variations in key regulators of serotonergic and noradrenergic systems comes to the conclusion that 5-HT is involved in top-down attentional control (Enge et al., 2014). In addition, dopamine and 5HT depletion (acute tryptophan or tyrosine/phenylalanin depletion) decreased performance in a Stroop interference task, indicating an increase in attentional control (Scholes et al., 2007).

#### Summary

5-HT has diverse functions in brain and body. Serotonergic signaling is also indicated in attention (Carter et al., 2005; Wingen et al., 2008), mainly in the processing of top-down attentional control (Scholes et al., 2007; Enge et al., 2011, 2014). However, information on how 5-HT influences voluntary and involuntary attentional mechanisms is limited and might demand further investigation.

### Oxytocin

#### General

Oxytocin is a nine amino acid neuropeptide, acting as a hormone and a neurotransmitter. It is synthesized in several nuclei of the hypothalamus (Mirtre et al., 2018). In the periphery, oxytocin has long been associated with lactation, osmoregulation and the regulation of energy balance (Ho and Blevins, 2013). However, oxytocin also plays an important role within the central nervous system, where it is implicated in social approach and maternal care behaviors, as well as in emotion processing (Yuan and Hou, 2015). Accordingly, oxytocin receptors appear to be more densely expressed in areas associated with emotion and social behaviors (e.g., amygdala, nucleus accumbens; Huber et al., 2005; Lee et al., 2009; Mirtre et al., 2018).

#### Effects of Oxytocin Modulation on Top-Down and Bottom-Up Visual Attention

A great amount of research has described oxytocin as an important neuromodulator of social and emotional processing (Yuan and Hou, 2015). In addition, it has been associated with cognitive functions such as the processing of sensory stimuli, social recognition, social memory and fear regulation (Ross and Young, 2009; Choe et al., 2015; Oettl et al., 2016; Jones et al., 2017; Grinevich and Stoop, 2018). Furthermore, oxytocin was found to influence selective attention when social stimuli were involved. Therefore, we have included it in this review.

For example, oxytocin modulated involuntary attention orienting to social stimuli (Puglia et al., 2018) and attentional allocation to social cues by influencing the interactions between brain networks associated with internal or external attentional control (Xin et al., 2018). These results are in line with other studies showing that oxytocin regulates visual attention and eye movements to external social stimuli (Domes et al., 2012, 2013; Clark-Elford et al., 2014). It was also hypothesized that oxytocin plays a prominent role in alerntness. Stoop (2012) proposed that vasopressin—a substance closely related to oxytocin—increases alert for external stimuli, while oxytocin decreases alert by modulating amygdala activity. Several studies indeed found a normalization of amygdala responses and attentional bias toward threat related cues after oxytocin application (Labuschagne et al., 2012; Clark-Elford et al., 2014). On the contrary, other studies have found an increase in attention toward emotional stimuli under oxytocin (Domes et al., 2013; Tollenaar et al., 2013; Clark-Elford et al., 2014; Xin et al., 2018). These discrepant results might be associated with natural variability in the DNA methylation of the oxytocin receptor gene, which has been linked to social attention capabilities (Puglia et al., 2018).

#### Summary

While ACh and DA are already established neuromodulators of attentional control, research has only recently begun to target the attentional effects of oxytocin. While the exact nature of these effects and their neural basis are not yet completely clear, it seems that oxytocin modulates visual selective attention to external social events (Domes et al., 2012, 2013; Clark-Elford et al., 2014).

### Glutamate

#### General

Although the neurotransmitters glutamate and gamma-aminobutyric acid (GABA) cannot be counted among brain neuromodulators, they fulfill important roles in brain functioning and cognitive processing. Together, these substances modulate the inhibitory-excitatory balance, which is crucial for cortical excitability and thus for normal brain functioning (Hampe et al., 2018). While the effects of GABA are mainly inhibitory, glutamate is the most important excitatory neurotransmitter in the brain. It exerts its influence on G protein-coupled metabotropic and ionotropic receptors that can be diveded into several subtypes (Vyklicky et al., 2014; Uno and Coyle, 2019). From these receptors, the ionotropic NMDA (N-Methyl-D-Aspartate) receptor type has been most extensively studied and is known to have important functions in synaptic transmission, plasticity and cogition (Collingridge et al., 2013; Kocsis et al., 2013; Vyklicky et al., 2014; Dauvermann et al., 2017; Valtcheva and Venance, 2019).

#### Effects of Glutamate on Top-Down and Bottom-Up Visual Attention

Several studies have found that subanesthetic doses of the NMDA receptor antagonist ketamine induce psychoses-like symptoms in healthy volunteers (Krystal et al., 1994; Malhotra et al., 1996). Ketamine influences memory (Malhotra et al., 1996; Morgan and Curran, 2006) and modulates attentional processes (Oranje et al., 2000; Watson et al., 2009; Gunduz-Bruce et al., 2012; Fuchs et al., 2015; von Düring et al., 2019; but see Morgan et al., 2004; van Wageningen et al., 2010). For example, Fuchs et al. (2015) investigated the influence of ketamine on top-down attentional control using a visual cueing paradigm. They found ketamine-induced impairments in voluntary attentional shifts to peripheral cues, but no drug effects on involuntary attentional cueing. Studies that examined the effect of ketamine on EEG data during visual or auditory attentional tasks generally demonstrate altered ERP amplitudes (P3: Oranje et al., 2000; Watson et al., 2009; Gunduz-Bruce et al., 2012; N2: Watson et al., 2009; N1: Oranje et al., 2000; MMN: Gunduz-Bruce et al., 2012). One study investigated the effect of low doses of ketamine on performance in a visual oddball task during simultaneous measuring of EEG, fMRI and electrodermal activity (Musso et al., 2011). The authors found decreased P3 amplitudes and BOLD responses under ketamine in regions commonly associated with selective attention. Thus, NMDA blockade affects early attentional processes as well as later voluntary control mechanisms (Watson et al., 2009). Schwertner et al. (2018) assumed that ketamine modulates EEG components by influencing stimulus discriminability (but see Rosburg and Schmidt (2018) for a different interpretation). This is in line with other research demonstrating that ketamine impairs the ability to both effectively gate the processing of sensory stimuli and process salient stimuli (Krystal et al., 1999; Umbricht et al., 2002). Thus, NMDA receptor functioning might play an important role in detecting changes in the environment, which is assumed to be central for the ability to orient toward salient novel stimuli. In this context, it is also regularly reported that ketamine has a strong influence on gamma-band oscillations (Thiebes et al., 2018; Curic et al., 2019), which seem to have an important role in bottom-up processing, perception and selective attention (Richter et al., 2017; Thiebes et al., 2018; Riddle et al., 2019).

#### Summary

The role of neurotransmitters in attention processing is less extensively studied than the roles of neuromodulators. However, there is increasing evidence that manipulation of the NMDA receptor with ketamine might have an effect on top-down (van Wageningen et al., 2010; Fuchs et al., 2015) and bottom-up attentional processing (Gunduz-Bruce et al., 2012). Most of this evidence comes from EEG studies demonstrating ketamine effects on several attention-related EEG components. In contrast to previous interpretations (Schwertner et al., 2018), Rosburg and Schmidt (2018) suggested that the reduced P3 amplitudes found after ketamine treatment are better explained by working memory impairments than by a drug-related increase in task difficulty due to alterations of perceived stimulus salience. On the other hand, the influence of ketamine on high frequency oscillations does suggest a connection between glutamate, bottom-up attentional processing and selective attention (Thiebes et al., 2018).

### GABA

#### General

While glutamate is the brain’s main excitatory neurotransmitter, the role of GABA in the mature brain appears to be mostly inhibitory. As with ACh, there are two main types of GABA receptors: an ionotropic GABA-A receptor and a metabotropic GABA-B receptor (Wu et al., 2016). While 20–30% of cortical neurons are GABAergic interneurons (Schmidt-Wilcke et al., 2018), the effects of GABA are not limited to inhibitory synaptic modulation. It is assumed to be involved in numerous cognitive functions (Schmidt-Wilcke et al., 2018; Kim et al., 2019). For example, GABA was found to influence working memory (Duncan et al., 2014; Yoon et al., 2016), impulsivity (Boy et al., 2011; Yoon et al., 2016), action selection (Steenbergen et al., 2015) and motor function (Stinear and Byblow, 2003; Zoghi et al., 2003; Duncan et al., 2014). On the other hand, GABA dysfunctions have been associated with several brain diseases (Schür et al., 2016; Kim Y.S. et al., 2017) and psychiatric disorders, such as schizophrenia (de Jonge et al., 2017), OCD (Li et al., 2019) or attention-deficit-hyperactiviy-disorder (ADHD; Edden et al., 2012).

#### Effects of GABA on Top-Down and Bottom-Up Visual Attention

Animal studies have found GABA to be involved in the regulation of attentional resources (Katzner et al., 2011; Paine et al., 2011; McGarrity et al., 2017) and to shape performance in visuo-spatial attention tasks (Petersen et al., 1987; Pezze et al., 2014). In humans, GABA seems to improve attentional selectivity in the visual cortex (van Loon et al., 2013; Sandberg et al., 2014), either by inhibiting irrelevant sensory information or by increasing the specifity of neural representation (Sandberg et al., 2014; Frangou et al., 2019). There is some evidence indicating that GABA might affect temporal aspects of attentional control more than spatial aspects (Kihara et al., 2016; Leonte et al., 2018). Furthermore, GABA can influence attentional processing through modulating cholinergic inputs. In rats, the infusion of positive and negative GABA modulators into the basal forebrain augments and decreases (respectively) the excitability of cholinergic projections, affecting performance in sustained attention tasks in both directions (Sarter, 1994; Moore et al., 1995; Sarter et al., 2001). This is in line with recent assumptions of GABA/ACh co-transmission (Saunders et al., 2015; Ma et al., 2018). In the study of Sandberg et al. (2014) participants with high GABA concentration in occipital areas (quantified by MR spectroscopy) reported to be better able to ignore irrelevant information. The authors speculated that a high GABA level does not automatically lead to more inhibition of bottom-up signals but that this GABAergic suppression can be influenced by top-down signals to match relevant behavioral goals. As such, high GABA levels would indicate the “potential strength of suppression between competing visual stimuli” (Sandberg et al., 2014).

#### Summary

In contrast to some neuromodulators, the evidence for GABA involvement in attentional processing seems to be quite solid. GABA was shown to shape visual attention by suppression of bottom-up signals and improving attentional selectivity in early visual areas (van Loon et al., 2013; Sandberg et al., 2014). These effects might be influenced by top-down signals (Sandberg et al., 2014) and implemented at least partly through modulation of cholinergic inputs (Saunders et al., 2015).

## Conclusion

In this review, we have discussed the importance and relative contributions of different neuromodulators to voluntary and involuntary visual attention. However, while the central roles of cholinergic, dopaminergic and noradrenergic systems in selective attention are widely accepted, there is less evidence concerning other neuromodulators, like serotonin or oxytocin. The same can be said about top-down and bottom-up attention. Whereas top-down processes are already subject to comprehensive investigations, the neuromodulatory mechanisms underlying bottom-up attention are less clear, as well as the neurochemical processes supporting a possible interaction of bottom-up and top-down systems. In general, dopamine and serotonin seem to be more closely associated with top-down attentional mechanisms, whereas acetylcholine, noradrenaline and oxytocin might also subserve specific roles in visual detection, salience processing and exogenous attentional orienting. The situation is complicated by the circumstance that the effects of neuromodulators in attentional processing seem to change depending on brain region, receptor subtype, level, task or release mode. For example, top-down and bottom-up effects might be driven by an interplay of cholinergic activity at muscarinic and nicotinic receptor types, with nACh receptors being more involved in modulating bottom-up attention and muscarinic receptor types being more involved in top-down attention. At the same time, the effects of most neuromodulators seem to vary across different brain regions. ACh might influence visual processing in primary visual cortex (via mACh receptors) and attention in higher cortical areas (via nACh receptors). Furthermore, the effect of NA was shown to increase or decrease PFC function depending on the different affinities of its adrenergic receptors for moderate or high neuromodulator levels. In addition, it was found that several neuromodulators show tonic and phasic firing modes, which might also influence their effects on attentional processing. Since bottom-up and top-down attention appear to be closely connected and neuromodulators have been shown to directly and indirectly interact (Avery and Krichmar, 2017), it is imaginable that some or even all of these processes work synergistically to shape human selective attention.

## Future Directions

Research about neuromodulatory effects on visual attention are complicated by the fact that attentional processes are hard to discriminate from working memory. Attention and working memory are closely intertwined and appear to be supported by overlapping mechanisms and brain regions (LaBar et al., 1999; Awh et al., 2006; Theeuwes et al., 2009; Gazzaley and Nobre, 2012). Therefore, it is not suprising that some neuromodulators have been shown to influence both processes (Arnsten and Goldman-Rakic, 1990; Sawaguchi and Goldman-Rakic, 1994; Gamo et al., 2010). Thus, future studies should be mindful of this interaction and try to include paradigms which are able to seperate working memory and attentional processes. Furthermore, including neuromodulators that are not yet extensively studied, such as oxytocin, might help gaining valuable insights about specific subcomponents of visual attention (in this case: social attention) and putting together a more complete picture of how visual attention is represented, modulated and expressed in the human brain. Another issue for future research might be the dissociation of attentional processes. It is still not sufficiently clear how different neuromodulators, their specific properties (e.g., receptor affinities, firing mode) or their neuronal interactions influence bottom-up and top-down control or the interplay of both processes. On top of that, recent studies have reported that the dichotomy of top-down and bottom-up attention seems to be obsolete and should be replaced by a more comprehensive model, that includes other factors such as reward and past selection history (Awh et al., 2012). For example, Hickey et al. (2010) showed that reward automatically triggered a selection bias toward a reward-associated feature, even when attending to that feature was opposed to the goals of the participant. Thus, there appear to be other influences on attentional selection than stimulus salience or voluntary intentions. In spite of these findings, there are still many open questions about the way reward affects the coordination of visual salience and voluntary attentional control (Feldmann-Wüstefeld et al., 2016), as well as concerning the underlying neurophysiological and neurobiological mechanisms. Since dopamine was shown to play a prominent role in reward processing, as well as in voluntary attention, this interaction might be an interesting starting point for future studies. Another promising area seems to be the investigation of brain oscillations. Several neuromodulators were found to influence brain oscillations during attentional processing. For example, ACh was shown to modulate gamma synchrony in rats (Howe et al., 2017) or alpha and beta oscillations in humans (Bauer et al., 2012). Additionally, DA could modulate PFC activity through phasic activiation of dopaminergic neurons that modify PFC activity and gamma oscillations (Lohani et al., 2019). This is important because neuronal communication is not just determined by anatomical connectivity and activity-dependent changes to the anatomical structure of the connection, but also by neuronal synchronization. Dynamic changes in synchronization might even be a core feature of cognition (Fries, 2015). Consequently, high-frequency gamma oscillations (30–100 Hz) were found to accompany various cognitive and psychological processes (Uhlhaas et al., 2011), coordinating activity within local circuits and between distant brain regions (Fries, 2015). It is assumed that oscillations in different frequency bands might subserve specific roles in selective attention. For example, gamma band oscillations seem to primarily carry out influences mediated by bottom-up projections, whereas influences mediated by top-down projections are carried out by alpha-beta band synchronization (Bastos et al., 2012). Interestingly, these top-down beta band influences are assumed to directly modulate bottom-up gamma band via cross-frequency interaction (Richter et al., 2017). In accordance with the development of new non-invasive stimulation methods (TMS, tDCS), that are specifically designed to target neural oscillations and the associated cognitive processes, the investigation of brain oscillations and their task-specific frequency profiles in connection with neurochemical modulation of different attentional processes (top-down, bottom-up, reward) seems to be a promising field for future research.

## Author Contributions

The order of authorship was a joint decision of the co-authors. Each author has participated sufficiently in the work to take public responsibility for the content. Authorship credit was based on substantial contribution to conception and design, execution, or analysis and interpretation of data. Both authors were involved in drafting the article or revising it critically for important intellectual content, read and approved the final version of the manuscript, and qualified for authorship.

## Conflict of Interest

The authors declare that the research was conducted in the absence of any commercial or financial relationships that could be construed as a potential conflict of interest.

## Abbreviations

5-HT: 5-hydroxytryptamine (serotonin)
ACh: acetylcholine
ADHD: attention-deficit-hyperactivity-disorder
BG: basal ganglia
BOLD: blood-oxygen-level-dependent
CHT: choline transporter
COMT: catechyl-O-methyltransferase
DA: dopamine
DAN: dorsal attention network
DAT: DA transporter
DBH: dopamine-beta-hydroxylase
DMN: default mode network
FCPN: fronto-parietal control network
FEF: frontal eye field
fMRI: functional magnetic resonance imaging
GABA: gamma-aminobutric acid
IPS: intraparietal sulcus
LC: locus coeruleus
mAChR: muscarinic ACh receptor
MRS: magnetic resonance spectroscopy
NA: noradrenaline
nAChR: nicotinic ACh receptor
NMDA: N-methyl -D-aspartate
PET: positron-emission-tomography
PFC: prefrontal cortex
SNR: signal-to-noise-ratio
SOA: stimulus-onset-asynchrony
tDCS: transcranial direct current stimulation
TMS: transcranial magnetic stimulation.
